# An Approach for Estimating Lightning Current Parameters Using the Empirical Mode Decomposition Method

**DOI:** 10.3390/s22249925

**Published:** 2022-12-16

**Authors:** Selma Grebović, Nermin Oprašić, Vahid Helać, Ivo Uglešić, Abdulah Akšamović, Samim Konjicija

**Affiliations:** 1 Faculty of Electrical Engineering, University of Sarajevo, 71000 Sarajevo, Bosnia and Herzegovina; 2 Faculty of Electrical Engineering and Computing Zagreb, University of Zagreb, 10000 Zagreb, Croatia

**Keywords:** Empirical Mode Decomposition (EMD), lightning current parameters, lightning measurement, lightning statistics

## Abstract

Lightning parameters are needed in different engineering applications. For the prediction of the severity of transient voltages in power systems, an accurate knowledge of the parameters of lightning currents is essential. All relevant standards and technical brochures recommend that lightning characteristics should be classified according to geographical regions instead of assuming that these characteristics are globally uniform. Many engineers and scientists suggest that better methods for lightning current measurements and analyses need to be developed. A system for direct lightning current measurements installed on Mount Lovćen is described in this paper. Observed data were analyzed, and statistical data on parameters that are of interest for engineering applications were obtained, as well as correlations between various lightning parameters. Furthermore, a novel approach for classifying and analyzing lightning data from direct measurements based on empirical mode decomposition (EMD) is proposed. Matlab was used as a tool for signal processing and statistical analysis. The methodology implemented in this work opens possibilities for automated analysis of large data sets and expressing lightning parameters in probabilistic terms from the data measured on site.

## 1. Introduction

With the continuous increase in the complexity of the electrical power system, growing exposure to the adverse effects of different environmental factors is further aggravating problems associated with reliability and safe operation of power system. Extreme weather phenomena and lightning in particular is one of the most common causes of faults and power supply interruptions. Systematic reviews of numerous observations of lightning events all around the world and information thus obtained provide valuable tools to reduce vulnerability and improve overall performance of power system. Damage to the power system is caused by both direct and indirect lightning strokes. In order to assess lightning effects and to design effective protection systems accurate lightning current parameters must be used. Lightning current parameters are of great importance in insulation coordination procedure, e.g., if this procedure is not properly determined the energy of lightning discharge can exceed energy handling capability of power system components [1,2,3].

Three approaches can be used to obtain lightning data: direct measurements using instrumented towers, direct measurements using the technique of the artificial initiation of lightning and lightning location systems [4,5]. Formatted lightning data from modern lightning location systems include: time and date of lightning stroke, GPS coordinates (2D), lightning current amplitude, lightning type, height (for inter-cloud lightning) and 2D statistical error [6,7]. Lightning location systems do not provide data about front and tail time of lightning current waveform. It is well known that front time has a high influence on insulation in power systems, while, for example, energy stresses of surge arresters strongly depend on tail time of the overvoltage wave [8,9,10,11]. Therefore, when conducting simulations of power system transients it is necessary to predict these data, as it is recommended in the CIGRE Brochure 549 (2013) and last IEEE review of parameters of lightning strokes (2005) [12,13].

In the field of lightning research for engineering applications, the most important data are obtained by the analysis of directly measured lightning current waveform. When designing such measuring systems, the first important step is the selection of a location with high lightning activity. Modern lightning location systems should be used for selection of regions with high lightning activity. Furthermore, it is important to correctly select the components of the measuring system, which will be in accordance with the specific characteristics of the lightning current that should be measured. In addition, it is necessary to develop tools for adequate and precise processing and analysis of measured data.

If a record of lightning current waveform is available, it is then possible, using appropriate numerical technique, to determine different parameters associated with that specific waveform. In every measurement, however, the measuring device is affected by environmental disturbances, referred to as noise, that alter characteristics of the output signal. Presence of noise can cause serious errors in measurement signal processing. In the case of lightning current measurement, in general, parameter determination may be difficult task due to the fact that all measured lightning current data are contaminated by considerable levels of noise, so additional processing steps must be undertaken in order to minimise effects of noise. The classification of recorded lightning current waveforms based on polarity and multiplicity is another important consideration in lightning studies. When dealing with a large amount of data from lightning monitoring systems, it is impractical to classify and analyze data manually. For such studies it becomes necessary to develop methodologies for automated classification and extraction of waveform parameters from the recorded data.

In the recent literature on lightning research, processing techniques are insufficiently considered. Several established methods are frequently reported: the Fourier transform, the short Fourier transform (STFT) [14], and the wavelet transform (WT) [15,16]. In [17], a time domain digital processing system for lightning current waveform parameters extraction is described. Using this approach procedure for parameters extraction from negative lightning flash with only one stroke was developed. In [18,19,20], the authors use Empirical Mode Decomposition (EMD) for discharge electric field pulse analyses, but in the recent literature, this method was not used for lightning current waveshape analyses. Therefore, this paper proposes a novel approach for analyzing lightning current waveform parameters and it is based on EMD. Continuing the work in [17], the authors expanded capabilities of previously developed signal processing procedure in terms of introducing new algorithm, increasing number of analysed features and including all typical types of discharges.

In this study, data from direct lightning current measurement system is analyzed using novel signal processing and parameter estimation technique and detailed statistics for one year observation period is presented. Correlations between various lightning parameters are established. This paper includes following contributions:

- a novel approach to lightning current waveform processing based on EMD for more accurate automatic lightning classification and lightning parameter extraction is introduced;
- statistical properties of lightning current parameters that are of great importance for engineering applications in region of mountain Lovćen (Montenegro) is presented;
- empirical expressions for cumulative peak current distributions of first and subsequent strokes are determined.

The rest of the paper is organized as follows. Section 2 introduces the types of lightning discharges and lightning current parameters. Description of observation site and lightning monitoring system is given in Section 3. Section 4 describes novel approach for lightning data analyses. Statistics of lightning current parameters and correlations between parameters are reported in Section 5 and the paper is concluded in Section 6.

## 2. Lightning Discharges and Lightning Parameters for Engineering Application

According to [4,21], cloud–to–ground lightning discharges are divided into four main types: upward or downward (by the direction of the motion of the initial leader) and positive or negative (by the sign of the charge deposited along the channel). Classification in [4] includes only “unipolar flashes” that transport charge of one polarity to ground. Lightning flashes that transport both negative and positive charges to ground called “bipolar flashes” are not included in this classification. More than one lightning stroke can hit the same place on the ground in short time interval. To identify number of strokes in a single flash the term multiplicity is introduced. Usually first strokes have larger currents than subsequent strokes that occur both in new and in previously formed channel. Further details on lightning phenomenon can be found in [4].

**Figure 1:**
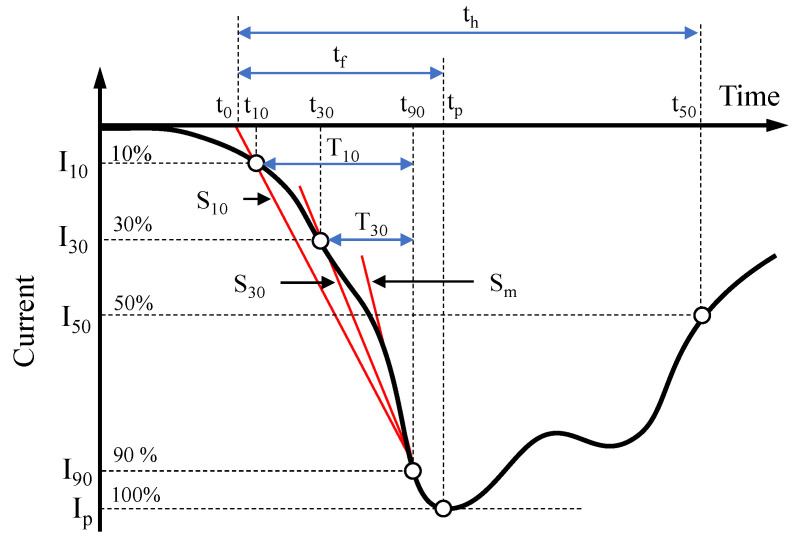
Typical lightning current waveshape and parameters, adapted from [22].

Different types of lightning discharges have different impacts on power systems. Therefore, it is very important to identify the parameters of lightning current. According to CIGRE publication [22], typical lightning current waveshape, shown in Figure 1 is characterized by a fast rising wavefront followed by slow subsequent decay. From engineering point of view, current front is the most significant part of current waveshape. Parameters that are commonly associated with current wavefront include:

- $I_{p}$ (kA): peak amplitude (highest current peak);
- $T_{10}$ (μs): interval between the 10% ($I_{10}$) and 90% ($I_{90}$) of the current peak on the current wavefront: $T_{10}=t_{90}-t_{10}$;
- $T_{30}$ (μs): interval between the 30% ($I_{30}$) and 90% ($I_{90}$) of the current peak on the current wavefront: $T_{30}=t_{90}-t_{30}$;
- $S_{10}$ (kA/μs): the average front steepness (rate of rise) between the 10% value point and 90% value point of the first peak: $S_{10}=\left(I_{90}-I_{10}\right)/T_{10}$;
- $S_{30}$ (kA/μs): the average front steepness (rate of rise) between the 30% value point and 90% value point of the first peak: $S_{30}=\left(I_{90}-I_{30}\right)/T_{30}$;
- $S_{m}$ (kA/μs): maximum current rate of rise on wavefront (maximum front steepness).

Based on the above parameters, the time duration of current front, $t_{f}$, is defined as time interval from $t_{0}$ to $t_{p}$ and is determined as shown in Figure 1. The time to half value, $t_{h}$, represents time interval from $t_{0}$ to the 50% value point of the first peak ($t_{50}$). The energy in a lightning flash is assessed generally by its charge, *Q*, defined as:

(1) Q=∫i(t)dt

and specific energy *E* is defined as:

(2) $$E=\int i^{2}\left(t\right)dt$$

## 3. Observation Site and Lightning Monitoring System

### 3.1. Location

**Figure 2:**
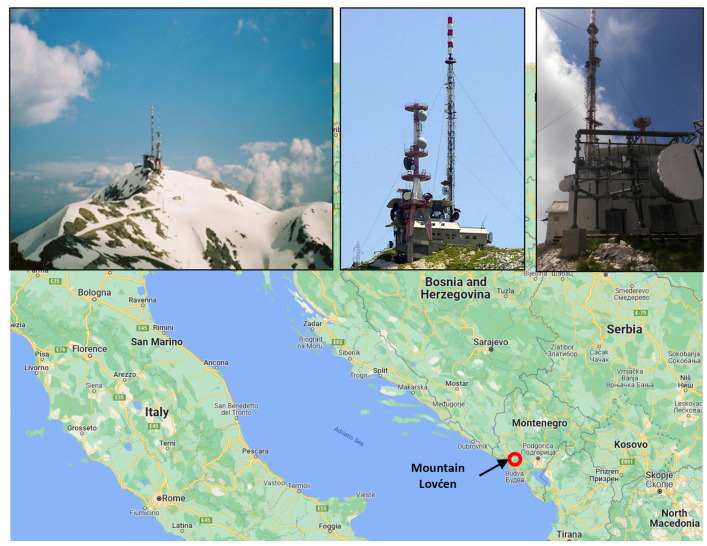
Observation site location.

Mountain Lovćen, with peak altitude of 1749 m is located in southwestern Montenegro, near the Adriatic Sea. Geographical location of mountain Lovćen and tower on which measuring equipment is installed are shown in Figure 2. Lightning current measurement equipment was installed on the 88 m high broadcasting tower, one of the most important communications hub in the region. The decision to install measurement equipment on this site was made based on previous reports and on data available from lightning location system (LLS).

LLS data have revealed that Lovćen, with 1063 strokes per square kilometer per year, has more than 100 times above median value in this region. Another contributing factor when choosing this site was the 500 kA maximum lightning current amplitude recorded by LLS reported in [23].

### 3.2. Measuring System

**Figure 3:**
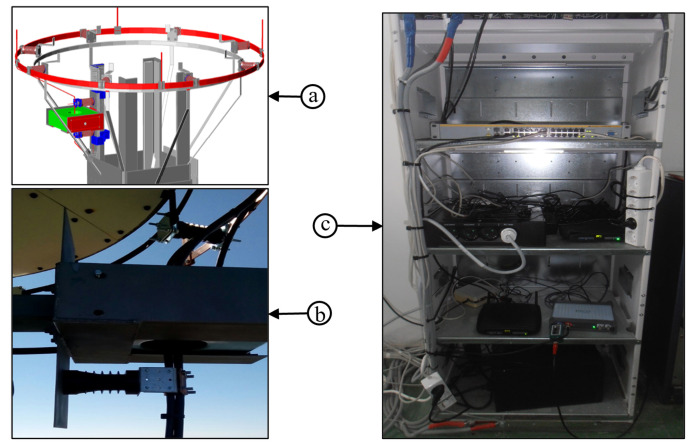
Measuring equipment on the site: (**a**) 3D view of the mounting of the current transformer, (**b**) current transformer, and (**c**) recording unit and power supply unit.

**Figure 4:**
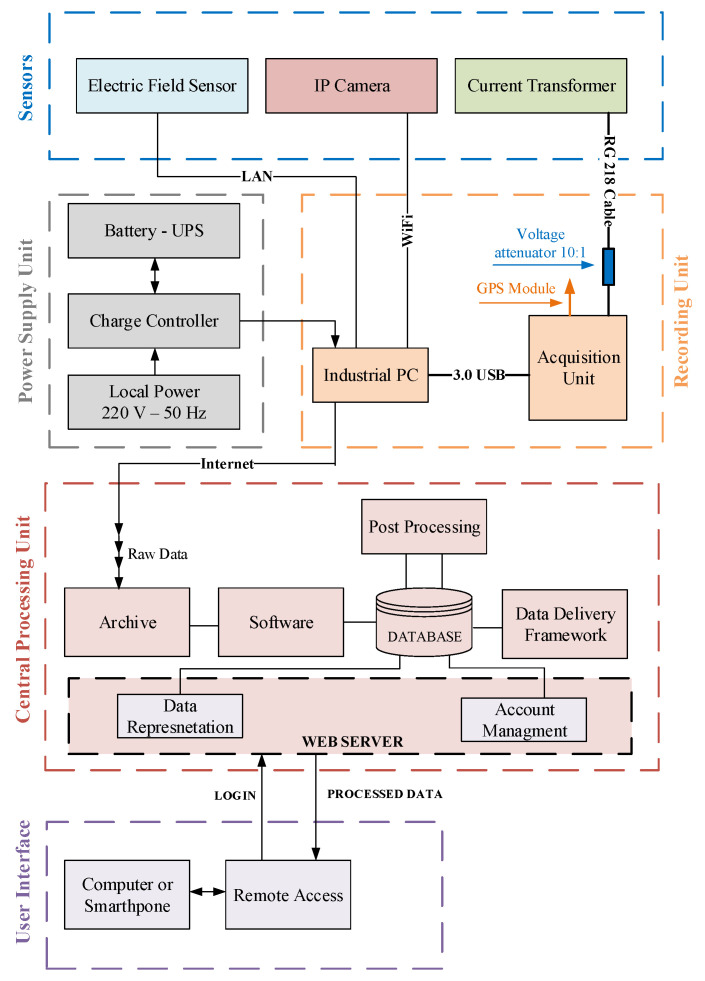
Block diagram of measuring system.

The lightning measurement system was constructed from a sensor, recording unit, power supply unit, central processing unit and user interface. A installed hardware is presented in Figure 3, while detailed block diagram of the system is shown in Figure 4. The sensor unit containing current transformer, electric field sensor and IP camera, was installed on tower top.

The lightning current sensor used was current transformer with 500 kA input range. Changes in electric field are registered using electric field sensor BOLTEK–EFM 100 Atmospheric Electric Field Monitor. IP camera (UFG1122 HD IP Camera) with 120 fps (frames per second), equipped with SD card and infrared cut filter for day/night operations.

The ecording unit is based on an industrial computer that records data from sensor unit. The acquisition unit is a four-channel card with an acquisition sampling rate of 8 MSa/s per channel and 15 bits vertical resolution.

Accurate timing is provided by an integrated GPS receiver. Local ethernet connection is used for communication with the remote server. Recording and processing units were installed inside the broadcasting tower and are supplied from AC mains. A low loss cable (RG218) was used to connect output from the current transformer to the input of acquisition unit. A voltage attenuator with the ratio 10/1 was installed at the acquisition card input. Data are transferred in real-time via internet to the central server and stored into the integral information system. Detailed information on system architecture is provided in [24].

## 4. Signal Processing and Parameter Estimation

Signals obtained directly from the measuring system contain considerable amount of noise. Important lightning current parameters can be distinguished without filtering directly from the measured current shape, but it is not possible to determine the exact values. Therefore, in order to extract the values of important parameters, it is necessary to apply the appropriate signal processing technique. To improve the parameter extraction process, empirical mode decomposition was introduced for lightning current waveform denoising.

### 4.1. EMD Algorithm and Parameters Determination

Empirical mode decomposition represents a method of breaking down a signal without leaving the time domain. This method is a powerful tool for analyzing natural signals, which are mostly non-linear and non-stationary. It serves as a signal processing technique based on an empirical and algorithm defined method. EMD can adaptively decompose a complex signal into a set of complete, almost orthogonal components. These components are known as Intrinsic Mode Functions (IMFs).

EMD filters out IMFs without requiring any preliminary understanding of the nature and quantity of the IMF components in the data. The main advantage of EMD compared with the widely used wavelet-based technique is that EMD can be used to decompose a signal without specifying the basics functions in advance, and the degree of decomposition is adaptively determined in accordance with the nature of the signal to be decomposed [20].

Due to its performance, EMD has been widely used in many disciplines. EMD was first proposed by Huang et al. in [25] and this approach is used in computational neuroscience, biomedical signal processing, climate signal analysis, audio signal processing, image processing, and seismic signal and discharge electric field pulse analyses [26]. Therefore, details of the EMD algorithm and denoising principles can be found in the literature [25,27,28,29].

**Figure 5:**
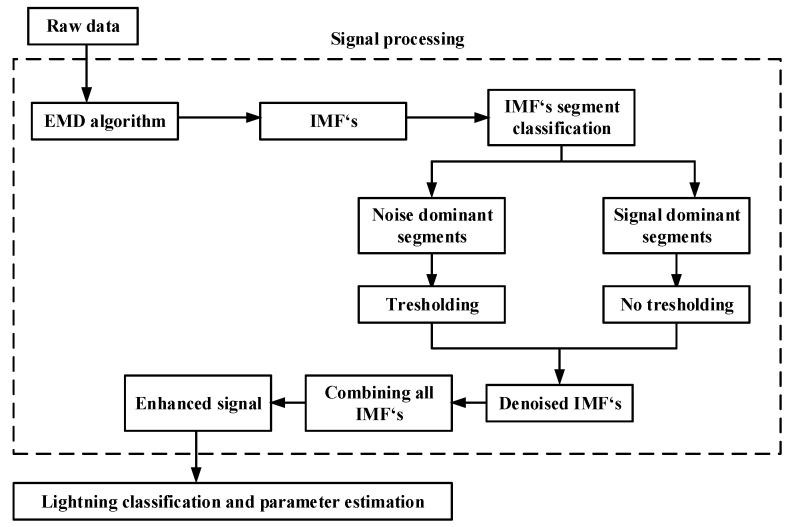
EMD-based adaptive lightning current enhancement and parameter determination system.

This paper introduces the EMD algorithm into analyses of lighting current waveforms for parameter determination. This study presents the concept of EMD and its application to lightning current signal processing. Figure 5 shows the proposed EMD-based adaptive thresholding lightning current enhancement concept.

The basic steps of proposed method are as follows:

- Step 1 Applying EMD algorithm to the raw data (noisy lightning current waveshape), which decomposes input signal in to IMFs.
- Step 2 IMFs segmentation into frames.
- Step 3 Frame classification into noise dominant and signal dominant frames.
- Step 4 Adaptive thresholding.
- Step 5 Combining of denoised IMFs.
- Step 6 Parameter determination from enhanced signal.

Proposed novel lightning current signal processing and parameter determination method was implemented in MATLAB.

### 4.2. Evaluation of Used Methodology

In order to evaluate accuracy of proposed lightning current parameters estimation procedure several experiments were conducted. The proposed processing method was applied to a set of three types of synthetic signals. Three types of standard CIGRE concave lightning current waveforms, with parameters given in Table 1, are generated with sampling rate of 8 Msamples/s. It was assumed that the measured lightning current can be represented at most in accordance to CIGRE concave lightning current model. In addition, it is assumed that the noise observed in measured signals is additive white Gaussian noise. These assumptions are reasonable due to fact that most of recorded lightning strokes are very similar to the assumed CIGRE model [12,13].

Large number of randomly generated synthetic signals were generated in Monte Carlo simulations that were used for evaluation of performance of proposed method. For each type of standard lightning current waveshapes from Table 1, 1000 synthetic signals with a signal-to-noise ratio (SNR) in the range of 0 to 25 dB were generated. These signals were then processed, using the proposed method described in Section 4.1; enhanced signals were obtained and subjected to classification and parameter estimation algorithms.

The difference between estimated parameters obtained from enhanced signals relative to original signal parameters (in Table 2) are listed in Table 2 and were used as criterion for performance evaluation.

From Table 2, it is clear that accuracy of some estimated parameters (peak current, tail time, total charge and specific energy) is almost independent of noise, while parameters such as front time and steepens are very sensitive to noise level. Estimated peak current values are almost constant in entire range of simulated SNR.

For peak currents greater than 10 kA, the average relative error was ±2.44% (from 0.81% to 6.65% with relative standard deviation below 7%). For lower peak currents (lower than 10 kA) average estimation error is slightly higher (±8.53%, with greatest error at 0 dB with value of 25.63%), and for SNR greater than 5 dB average estimation error was ±5.11%. These results suggests that proposed procedure can estimate with high accuracy peak current values in wide range of SNR (from 5 dB to 25 dB). As expected, for low current amplitudes and for SNR below 5 dB accuracy is decreasing.

As it can be seen from Equations (1) and (2), total charge and specific energy are functions of lightning current and due to this fact these parameters are also estimated with high accuracy within entire region of simulated SNR with average relative error of ±2.84% and ±0.77%, respectively. Noise level does not significantly affect these parameters since integration, in principle, represents a low pass filter. Time duration and steepness parameters estimation, however, are in general more variable and more sensitive to SNR. Tail time duration is estimated with the average relative error of ±3.55%.

Waveform parameters front time and steepness in the investigated range of values and noise levels are estimated with higher average relative errors of 34.99% and 16.71%, respectively. The estimation of these parameters is significantly affected by the noise level. For fast rising currents ($t_{f}$ around 1 μs) in extreme case (at SNR = 0 dB), estimation errors may be up to 200%, and in this case, estimation is not reliable. However, in the more common range of SNR values (>10 dB), the average relative error for the front time is ±15.04%, while for the steepness, it is ±7.32%. Considering the standard tolerances given in [30,31] (for front time ±30% and for tail time ±20%), the obtained results are within the acceptable range.

## 5. Results of Observation

During the observation period of one year, 163 lightning events were recorded. Using the developed approach for automated data analysis, different types of lightning discharges were identified. The total number of lightning flashes was 64. The analyzed events were classified as given in Table 3. Detailed statistics were performed only for negative strokes due to the representative sample size.

### 5.1. Statistical Distribution of Lightning Parameters

It is generally agreed that the statistical distribution of lightning parameters follows the log-normal distribution, where the statistical variation of the logarithm of a random variable, *x*, follows the Gaussian distribution. The log-normal probability density function, p(x), is defined as in [13]:

(3) $$p\left(x\right)=\frac{1}{\sqrt{2}\pi x\sigma_{lnx}}e^{-\frac{1}{2}{\left(\frac{lnx-lnx_{m}}{\sigma_{lnx}}\right)}^{2}}=\frac{1}{\sqrt{2}\pi x\sigma_{lnx}}e^{-u^{2}}$$

where $\sigma_{lnx}$ is standard deviation of lnx, and $x_{m}$ is median value of *x*.

Therefore, $x_{m}$ and $\sigma_{lnx}$ need to be known to estimate the statistical distribution of a lightning parameter. The cumulative probability, $P_{c}\left(x\right)$, that the parameter will exceed *x*, is given by integrating Equation (3) between $u_{0}$ and *∞*, resulting in:

(4) $$P_{c}\left(x\right)=\frac{1}{\sqrt{\pi }}\int_{u_{0}}^{\infty }e^{-u^{2}du}=\frac{1}{2}erfc\left(u_{0}\right)$$

For approximating the log-normal distribution $P_{c}$ of lightning current peak, a simplified equation given by Anderson in [22,32] is also used:

(5) $$P_{c}\left(I>I_{p}\right)=\frac{1}{1+{\left(\frac{I_{p}}{\mu }\right)}^{\rho }}$$

where μ and ρ are calculated from empirical data.

Various correlations among lightning parameters have been found [13]. Assuming log-normal distributed random variables *x* and *y*, relationship between *x* and *y* can be expressed as:

(6) $$y=ax^{d}$$

### 5.2. Negative Flashes

**Figure 6:**
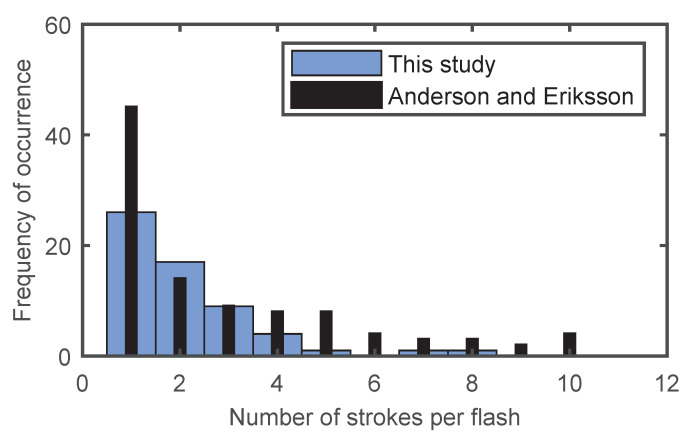
Statistical distribution of multicomponent negative lightning flashes.

As can be noted from Table 3, 59 first negative strokes and 86 subsequent negative strokes were analyzed. For the purpose of such analysis novel proposed processing method was used. The statistical distribution of multicomponent lightning flashes recorded in this study and compared with Anderson and Ericson (in [22]) is given in Figure 6. The frequency of the occurrence of multicomponent flashes in this region is very similar to that of Anderson and Ericson which is widely accepted.

**Figure 7:**
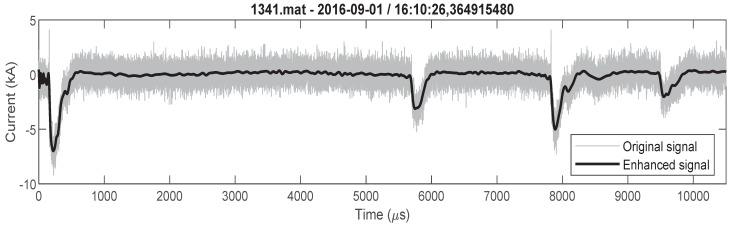
Negative multicomponent lighting flash (flash consists of four strokes) original and enhanced signal.

Classification, analysis and parameter determination are more challenging tasks for lightning flashes that consists of more stokes than for lightning flashes with single stroke. Therefore, as an example, a multicomponent lightning flash that consists of four negative strokes is presented in Figure 7. Figure 7 shows the originally measured signal and enhanced signal. Determined parameters that are important for engineering applications are presented in Table 4.

Statistical parameters resulting from the measurements from this study during observation period of one year are given in Table 5 and Table 6. First and subsequent negative currents were considered. After the log transformation, the Lilliefors test for normality, considering the 95% level of significance, was applied for the complete data set. It proved to be significant for most parameters of first negative strokes, similarly to the results presented by Anderson and Ericson in [22,33].

**Figure 8:**
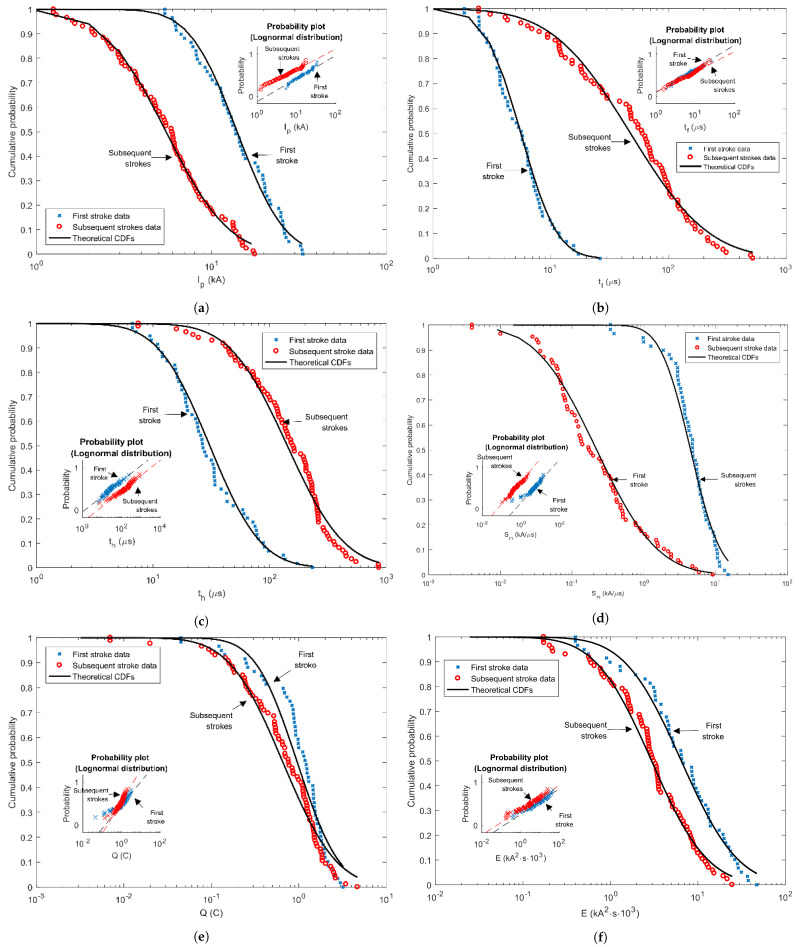
Cumulative statistical distributions of first and subsequent negative stroke currents—measured data and theoretical CDFs: (**a**) Peak stroke currents, (**b**) Front times, (**c**) Tail times, (**d**) Maximum steepness, (**e**) Stroke charges, (**f**) Specific energies.

Cumulative statistical distributions of various parameters for the first and subsequent strokes are presented in the figures below (from Figure 8), as well as probability plots for lognormal distribution. From the figures, it can be seen that the measured data for the first and subsequent negative stokes are in good agreement with the theoretical cumulative distribution function. As indicated by the *p*-value from Table 5 at a significance level of 95%, it can be concluded that most of the analyzed parameters of the first negative strokes are distributed according to log-normal distribution. The total charge and maximum steepness for first negative strokes, as well as most parameters of the subsequent strokes, are similarly distributed, but failed the Lillifors test and, therefore, the log-normal distribution of these parameters cannot be confirmed. Very important formulas for cumulative probability as a function of the peak current for the first and subsequent stokes are performed from these results. The approximate expressions for cumulative probability of first and subsequent negative strokes current are given by Equations (7) and (8), respectively.

(7) $$P_{c}\left(I>I_{p}\right)=\frac{1}{1+{\left(\frac{I_{p}}{14.05}\right)}^{3.404}}$$

(8) $$P_{c}\left(I>I_{p}\right)=\frac{1}{1+{\left(\frac{I_{p}}{5.51}\right)}^{2.597}}$$

It is well known that these expressions have direct application in assessment of the lightning performance of electrical systems especially in insulation coordination studies. Therefore, it is of great importance to develop such formulas for different regions worldwide.

### 5.3. Correlations between Parameters for First Stoke in Negative Flashes

Correlations between various parameters of recorded lightning current waveshapes are considered by using fitting curves given by Equation (6) or it can be also represented with equation:

(9) ln(y)=ln(a)+dln(x)

Correlation coefficients among parameters are given in Table 7. Results indicated that correlations are observed between almost all parameters. Few correlations that are not significant are marked in tables. Based on *p*-values from Table 8 statistically significance of correlations are confirmed. Strongest correlations were observed between peak current and all other parameters except tail time. Table 9 presents correlation expressions (described with function given in (9)) along with correlation coefficients *r* for such parameters, following logarithmic linear regression.

**Figure 9:**
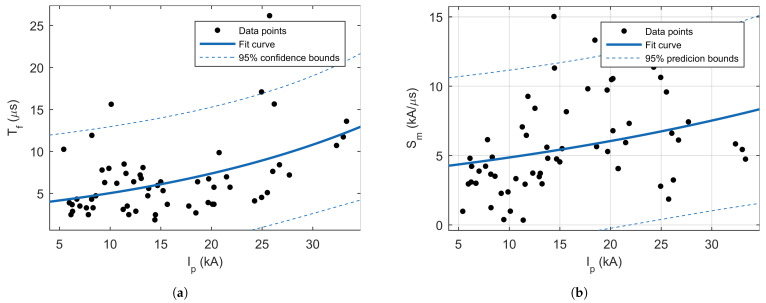
Scatterplots of the relations: (**a**) between first negative peak current and current front time, and (**b**) between first negative peak current and maximum steepness.

**Figure 10:**
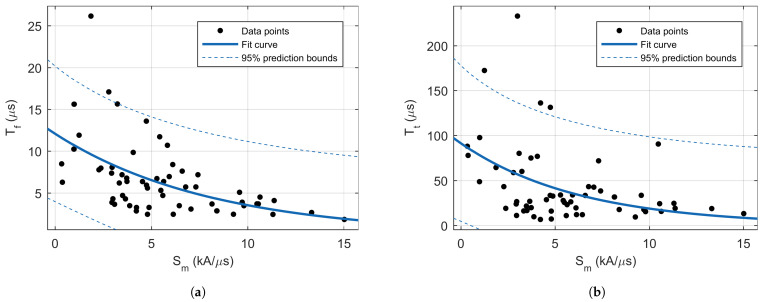
Scatterplots of the relations: (**a**) between maximum steepness and current front time, and (**b**) between maximum steepness and current tail time.

Similarly, as it is published in the literature, in this study, a correlation between peaks current and front time was observed. Additionally, a similar correlation exists between the peak current and maximum steepness (see Figure 9), with a similar correlation coefficients in available literature. As expected, a very strong correlation was observed between the peak current and total charge and specific energy. An interesting observation is that both the front and tail time are negatively correlated with steepness (see Figure 10).

### 5.4. Positive Flashes

**Figure 11:**
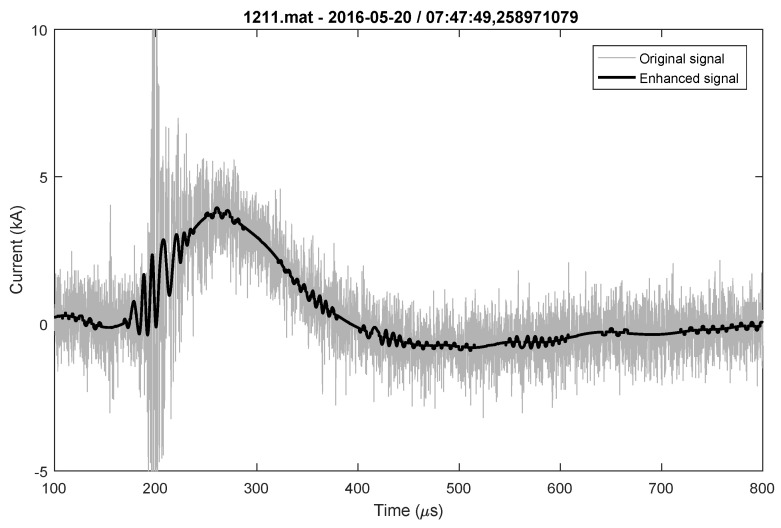
Positive lighting flash original and enhanced signal.

Four positive lightning flashes were observed, each with single stroke. The parameters for positive flashes are given in Table 10. As an example, the original and enhanced positive stroke are shown in Figure 11.

### 5.5. Bipolar Flashes

**Figure 12:**
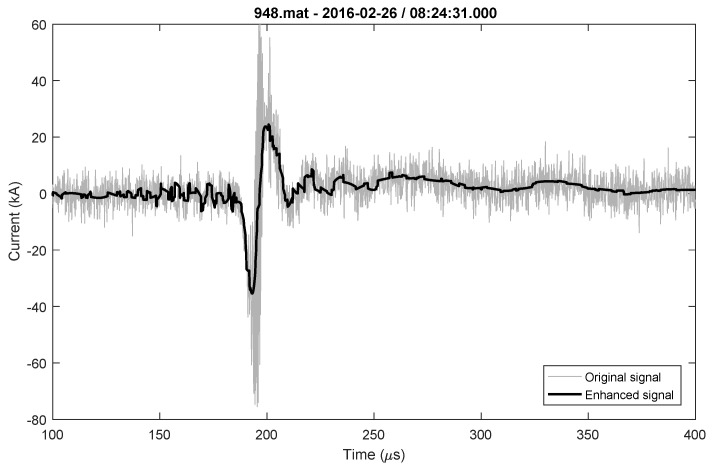
Bipolar lighting flash original and enhanced signal.

During the observation period of one year, one bipolar lightning flash was recorded on the 26th of February 2016, and it is presented in Figure 12. The parameters for the bipolar flash are given in Table 11. All parameters were determined for the positive and negative parts of the lightning current waveshape.

## 6. Conclusions

This paper presents the statistics of lightning current parameters obtained by processing the data collected by direct measurement at the broadcasting tower on Mount Lovćen. The analyzed data were collected during the one-year observation period. A new EMD-based approach for more accurate lightning classification and lightning parameter extractionwas applied to the data analysis. The introduction of the EMD algorithm significantly improved the accuracy of determining lightning current parameters compared to the methods previously used by the authors. Unlike conventional filters, using this algorithm in the proposed scheme, the phase shift of the signal is almost eliminated.

Based on the cumulative distributions of the peak current of the first and subsequent strokes, the formulas for determining the probability of the occurrence of the peak current and the expression relating the peak current and other important parameters were generated. These expressions can be used directly in power system analyses. The statistical data for this region showed that most of the parameters for the first negative stroke are distributed according to the lognormal distribution and are very similar to their representation in contemporary literature. This study also confirmed that most of recorded events are negative lightning strokes, while positive and bipolar lightning strokes are rare. However, positive and bipolar lightning flashes are very dangerous, especially bipolar flashes (which transmit a large amount of energy to the ground), and should be taken into account when designing lightning protection.

Many uncertainties regarding lightning events presently exist, and therefore better methods for lightning current measurements and waveshape analyses should be developed. It should be continued with efforts to collect data for formulation of lightning parameters according to geographical regions and for developing important formulas for power system lightning protection as well as developing correlation expressions among lighting parameters.

For the signal processing methodologies used, it was shown that the accuracy of determining the front time and steepness should be improved. For this improvement, better acquisition units should be installed in the measurement system, for example, with a much higher sampling rate (50 MSa/s or 100 MSa/s) in order to be able to better record front time that has a very short duration (from 1 to several microseconds). In the future, the dynamic characteristics of the measurement system should be taken into account when analyzing the signal-to-noise ratio in order to further improve the signal processing.

## Figures and Tables

**Table 1:** Typical lightning current waveshape parameters.

	$I_{\max}$(kA)	$t_{f}\left(\mu s\right)$	$t_{t}\left(\mu s\right)$	$S_{m}\left(\mathrm{kA}/\mu s\right)$	Q(C)	$E({\mathrm{kA}}^{2}s\cdot 10^{3})$
$I_{1}$	100	1.2	50	150	7.0	350
$I_{2}$	31	3.0	75	20	3.0	50
$I_{3}$	10	1.0	100	20	1.5	7

**Table 2:** The Monte Carlo simulations for estimation of parameter values.

SNR (dB)	$I_{\max}\left(\mathrm{kA}\right)$	$t_{f}\left(\mu s\right)$	$t_{t}\left(\mu s\right)$	$S_{m}\left(\mathrm{kA}/\mu s\right)$	Q(C)	$E({\mathrm{kA}}^{2}s\cdot 10^{3})$
CIGRE concave shape $I_{1}$						
0	98.74 ± 7.16	1.9610 ± 0.2407	49.664 ± 4.157	91.56 ± 10.30	6.97 ± 0.12	368.90 ± 6.72
5	98.56 ± 7.08	1.7498 ± 0.1494	49.664 ± 4.194	107.48 ± 8.11	7.03 ± 0.10	355.74 ± 5.06
10	98.42 ± 7.07	1.5492 ± 0.1494	51.001 ± 4.185	116.45 ± 8.94	6.89 ± 0.02	352.49 ± 0.92
15	98.33 ± 7.06	1.6791 ± 0.1660	49.664 ± 4.192	151.70 ± 5.08	6.90 ± 0.01	351.93 ± 0.84
20	98.37 ± 7.07	1.4790 ± 0.1328	49.664 ± 4.185	148.94 ± 1.44	6.90 ± 0.01	352.44 ± 0.15
25	97.00 ± 6.99	1.3198 ± 0.1327	49.664 ± 4.352	147.59 ± 1.53	6.90 ± 0.01	351.77 ± 0.19
CIGRE concave shape $I_{2}$						
0	32.89 ± 1.32	3.5201 ± 0.3517	66.564 ± 8.731	13.06 ± 2.16	3.07 ± 0.19	50.14 ± 1.10
5	32.12 ± 0.98	2.9164 ± 0.3184	70.912 ± 6.386	15.44 ± 3.05	3.07 ± 0.12	49.61 ± 0.66
10	32.07 ± 0.07	2.9716 ± 0.2633	71.283 ± 5.253	16.84 ± 4.32	3.04 ± 0.09	49.24 ± 0.22
15	31.49 ± 0.03	2.8612 ± 0.2794	72.826 ± 5.411	18.86 ± 0.68	3.05 ± 0.09	49.24 ± 0.16
20	31.67 ± 0.04	2.7784 ± 0.2619	75.167 ± 5.282	19.12 ± 2.36	3.04 ± 0.07	49.30 ± 0.09
25	31.40 ± 0.04	2.7048 ± 0.2664	75.637 ± 5.378	20.31 ± 1.53	3.04 ± 0.04	49.35 ± 0.02
CIGRE concave shape $I_{3}$						
0	12.55 ± 2.56	2.8669 ± 0.3912	89.631 ± 16.965	10.14 ± 7.41	1.38 ± 0.11	7.08 ± 0.37
5	10.93 ± 0.95	2.4818 ± 0.2226	90.224 ± 9.367	12.24 ± 8.71	1.41 ± 0.05	7.11 ± 0.15
10	10.82 ± 0.83	1.3201 ± 0.1874	91.638 ± 7.963	17.77 ± 5.44	1.43 ± 0.05	7.05 ± 0.05
15	10.54 ± 0.76	1.1898 ± 0.1217	92.348 ± 7.245	18.70 ± 6.39	1.48 ± 0.02	7.04 ± 0.03
20	9.86 ± 0.77	1.1617 ± 0.1323	97.891 ± 7.751	18.49 ± 5.72	1.48 ± 0.02	7.03 ± 0.03
25	9.89 ± 0.59	1.0973 ± 0.0984	99.654 ± 7.778	18.12 ± 4.38	1.48 ± 0.02	7.01 ± 0.03

**Table 3:** Observed lightning events.

Type	Number of Samples
First negative stroke	59
Subsequent negative strokes	86
First positive strokes	4
Bipolar strokes	1

**Table 4:** Parameters for multicomponent negative flash from Figure 7.

No. Strokes	$I_{p}\left(\mathrm{kA}\right)$	$t_{f}\left(\mu s\right)$	$t_{t}\left(\mu s\right)$	$S_{m}\left(\mathrm{kA}/\mu s\right)$	Q(C)	$E({\mathrm{kA}}^{2}s\cdot 10^{3})$
1	−8.343	63.565	189.824	0.625	−2.190	9.379
2	−4.193	63.433	222.976	0.315	−1.505	3.530
3	−6.180	70.210	185.600	0.425	−1.864	5.590
4	−2.988	66.067	327.936	0.215	−1.453	2.282

**Table 5:** Statistical parameters of measured first negative strokes values.

Parameter	Min	Max	Geometric Mean	Median	μ	$\sigma_{\mathrm{lnx}}$	*p*-Value	Lillifors Test (α = 0.05)
$I_{p}$ (kA)	5.424	33.335	14.041	13.806	2.642	0.4952	0.2521	TRUE
$t_{f}$ (μs)	1.834	26.162	5.5564	5.718	1.715	0.5618	0.5000	TRUE
$t_{t}$ (μs)	6.656	232.896	30.622	26.816	3.422	0.8027	0.0710	TRUE
$S_{m}$ (kA/μs)	0.344	15.018	4.481	4.796	1.499	0.7593	0.0088	FALSE
*Q* (C)	0.045	3.198	0.961	1.215	0.039	0.8692	0.0010	FALSE
*E* (kA${}^{2}$s$\cdot 10^{3}$)	0.398	47.086	6.467	6.808	1.867	1.1753	0.3895	TRUE

**Table 6:** Statistical parameters of measured subsequent negative strokes.

Parameter	Min	Max	Geometric Mean	Median	μ	$\sigma_{\mathrm{lnx}}$	*p*-Value	Lillifors Test (α = 0.05)
$I_{p}$ (kA)	1.254	17.720	5.508	5.987	1.706	0.6507	0.4272	TRUE
$t_{f}$ (μs)	2.446	521.856	47.620	61.877	3.863	1.2103	0.0102	FALSE
$t_{t}$ (μs)	7.488	865.152	141.589	160.672	4.953	0.8894	0.0166	FALSE
$S_{m}$ (kA/μs)	0.400	9.032	0.2202	1.770	1.513	1.5377	0.0483	FALSE
*Q* (C)	0.007	4.684	0.658	0.773	0.418	1.1135	0.0079	FALSE
*E* (kA${}^{2}$s$\cdot 10^{3}$)	0.173	24.652	2.961	3.204	1.161	2.9795	0.0393	FALSE

**Table 7:** Correlation coefficients between lightning parameters.

	$\ln(I_{p})$	$\ln(T_{f})$	$\ln(T_{t})$	$\ln(S_{m})$	ln(Q)	ln(E)
$\ln(I_{p})$	1	0.3916	0.0546	0.4294	0.6522	0.8513
$\ln(T_{f})$	0.3916	1	0.4626	−0.5404	0.3076	0.5042
$\ln(T_{t})$	0.0546 *	0.4626	1	−0.4353	0.4588	0.4681
$\ln(S_{m})$	0.4294	−0.5404	−0.4353	1	0.1889	0.2124
ln(Q)	0.6522	0.3076 *	0.4588	0.1889 *	1	0.8675
ln(E)	0.8513	0.5042	0.4681	0.2124	0.8675	1

* Corrrelation not statisticaliy significant.

**Table 8:** *p*-values.

	$\ln(I_{p})$	$\ln(T_{f})$	$\ln(T_{t})$	$\ln(S_{m})$	ln(Q)	ln(E)
$\ln(I_{p})$	1.0000	0.0022	0.6810	0.0007	0.0000	0.0000
$\ln(T_{f})$	0.0022	1.0000	0.0002	0.0000	0.0178	0.0000
$\ln(T_{t})$	0.6810 *	0.0002	1.0000	0.0006	0.0003	0.0002
$\ln(S_{m})$	0.0007	0.0000	0.0006	1.0000	0.1519	0.1063
ln(Q)	0.0000	0.0178 *	0.0003	0.1519 *	1.000	0.0000
ln(E)	0.0000	0.0000	0.0002	0.1063	0.0000	1.0000

* Corrrelation not statisticaliy significant.

**Table 9:** Correlation coefficients for peak current and other lightning parameters.

	a	d	r
** $T_{f}\left(I_{p}\right)$ **	1.7182	0.4443	0.3916
** $S_{m}\left(I_{p}\right)$ **	0.7868	0.6585	0.4294
** $Q(I_{p})$ **	0.0467	1.1449	0.6522
** $E(I_{p})$ **	0.0311	2.0206	0.8513

**Table 10:** Parameters for recorded positive flashes.

No. Stroke	$I_{p}\left(\mathrm{kA}\right)$	$t_{f}\left(\mu s\right)$	$t_{t}\left(\mu s\right)$	$S_{m}\left(\mathrm{kA}/\mu s\right)$	Q(C)	$E({\mathrm{kA}}^{2}s\cdot 10^{3})$
1	1.970	139.353	233.664	0.028	0.657	0.664
2	2.035	58.823	221.952	0.066	0.616	0.629
3	4.587	67.137	152.192	0.128	0.935	2.148
4	3.114	50.655	171.136	0.116	0.810	1.346

**Table 11:** Parameters for recorded bipolar flash.

Polarity	$I_{p}\left(\mathrm{kA}\right)$	$t_{f}\left(\mu s\right)$	$t_{t}\left(\mu s\right)$	$S_{m}\left(\mathrm{kA}/\mu s\right)$	Q(C)	$E({\mathrm{kA}}^{2}s\cdot 10^{3})$
Negative	−35.376	4.313	−15.792	−0.375	0.657	11.411
Positive	36.512	3.520	19.016	1.162	0.616	11.411

## Data Availability

The data that support the findings of this study are available from the corresponding author upon reasonable request.

## Abbreviations

The following abbreviations are used in this manuscript:

LLS	Lightning Location System
EMD	Empirical Mode Decomposition
IMF	Intrinsic Mode Function
SNR	Signal-to-Noise Ratio
STFT	Short Fourier Transform
WT	Wavelet Transform
CDF	Cumulative Distribution Function
